# Symptoms of psychosis, depression, and suicide ideation among individuals in a first episode of psychosis: The mechanistic role of clinical insight and cognitive functioning

**DOI:** 10.1192/j.eurpsy.2021.1412

**Published:** 2021-08-13

**Authors:** L. Bornheimer, J. Wojtalik, J. Li, D. Cobia, M. Smith

**Affiliations:** 1 Department Of Psychiatry And School Of Social Work, University of Michigan, Ann Arbor, United States of America; 2 School Of Applied Social Sciences, Case Western Reserve University, Cleveland, United States of America; 3 School Of Social Work, University of Michigan, Ann Arbor, United States of America; 4 Social Work, Brigham Young University, Provo, United States of America

**Keywords:** insight, Suicide ideation, psychosis, Depression

## Abstract

**Introduction:**

First-episode psychosis (FEP) is a particularly high-risk period in which risk for suicide death is elevated by 60% as compared to individuals in later stages of psychotic illness. Clinical insight and cognition have been studied in schizophrenia in relation to suicide ideation and attempt, yet, less is understood within the context of early-phase of illness and FEP.

**Objectives:**

This study examined whether clinical insight and cognitive functioning served as a mechanism in the relationships between depression, positive symptoms, negative symptoms, and suicide ideation over time among individuals in FEP.

**Methods:**

Data were obtained from the Recovery After an Initial Schizophrenia Episode (RAISE) project. Participants (n=404) included adults in FEP between ages 15 and 40. Structural equation modeling was used in Mplus8 to examine the proposed mediation model.

**Results:**

**Figure:**
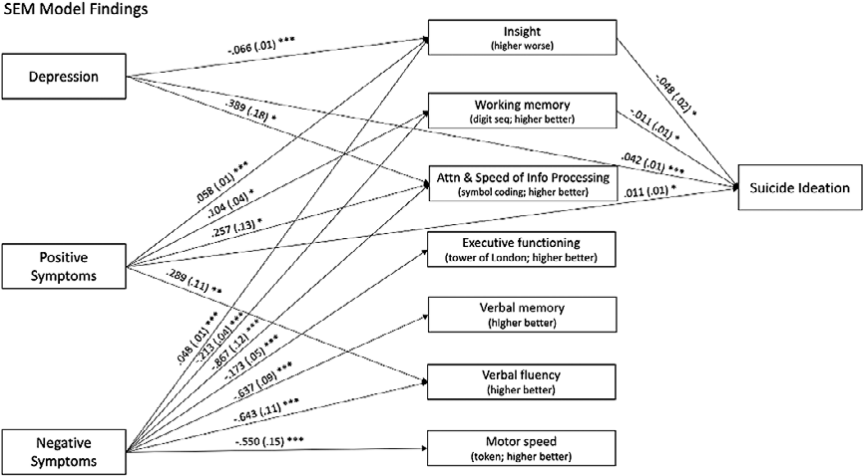

Clinical insight and working memory functioned as mechanisms in the relationships between depression, positive symptoms, negative symptoms, and suicide ideation. As depression decreased and positive and negative symptoms increased, clinical insight was shown to be poorer, which in turn related to decreased suicide ideation. As positive symptoms increased and negative symptoms decreased, working memory was shown to be stronger, which in turn related to decreased suicide ideation.

**Conclusions:**

Implications surround the importance of cognitive testing and approaches aiming to strengthen cognitive functioning given the relationships between cognition and suicide ideation in FEP. Also, of importance, it is imperative practitioners have awareness of the insight paradox given the complex and dynamic relationships between clinical insight and suicide thoughts and behaviors.

